# The Possibilities of Medicine

**Published:** 1891-08

**Authors:** 


### THE POSSIBILITIES OF MEDICINE.

At the recent commencement exercises of Long Island College Hospital Professor T. Gailliard Thomas, in giving the concluding address, spoke as follows:

Even in these unsentimental days many men chose their occupations more because of attending dangers and chances of heroism than from any less romantic notion of their usefulness. Thus the youth of a smiling country are persuaded to risk their lives on the sea, and the painter in the hope of fame starves at his work in the attic. Young men would willingly lay down their lives for the glory of a Sheridan or a Farragut. Arms, literature, art, law, divinity, all had bright and glorious rewards for those who had the courage to aspire to them and patience to suffer for them, the industry to work for them and the genius to obtain them. Think you, continued the speaker, that the science of medicine, founded 400 years before the birth of Christ the chosen work of our Saviour himself, the most far reaching and benign of all modern pursuits, stands alone in its inability to reward its votaries? Far from it. Look back with me into history, and I shall not be called upon to take you into the fields of ancient history, even, and it will go hard but I shall make you agree with me that of all callings, all pursuits, all professions, the rewards of medicine are greater, more lofty, more desirable and more enduring than those of any other in existence. His hearers would agree that he who had done the greatest good for his fellowman had, in the doing of, won the greatest rewards in earth's posessions, even if no mortal man knew of the deed but him. The forces of civilization work hand in hand for the common good. All the forces of intelligence, law, the fine arts religion and medicine were all contributing their quota. Hygiene—the science of cleanliness—as taught by medicine, had wiped off the globe the terrible scourges which made the dark ages so terrible. Cholera, the dread monster, had been almost totally shut up in its birthplace in Asia and checked in its wanderings over the world. Quarantine had transferred yellow fever from a universal menace into a local distemper. Smallpox had been lashed to hell by the agent vaccination, and like a whimpering hound was held securely in leash. The work of medicine had far more numerous conquests than even those enumerated. So wonderful, so startling, so extraordinary were those results that he feared his non-medical hearers would suspect him of boasting if he read the full record of the proud achievements of medicine during the past hundred years. But conquests in the past in no wise curtailed the possible achievements in the future. One great initial discovery ever opened the way for numberless others equally as great to be made. This was illustrated by the microscope, by the telescope, by the circulation of the blood, by steam and electricity. The discovery of the transmission of disease by bacilli had brought the students of to-day upon a plane far more elevated than that which even the youngest of their teachers occupied upon his graduation. The present students' possibilities in medicine were proportionately greater than his were, and it was their function to profit by their good fortune.

The speaker declared if he had the power to accomplish one wish in his life he thought he would select the destruction of the process by which alcohol was created. If that were denied him the power of stamping out forever those contagious diseases which fill our graves with curly heads and dimpled cheeks and our homes with sorrow that knows no comforting. He would destroy those terrors of the household, scarlatina, diphtheria and the host of contagious maladies which went hand in hand with them. The first of these wishes was impossible of attainment. But not so of the second. The way of its accomplishment was open to every man with willing hand, determined mind and intelligent brain. Surely it was not too sanguine a prediction that the next century might see the extinction of contagious disease. Elaborating this point, the speaker pointed that since 1799, the date of Jenner's discovery of vaccination for small-pox —and unless the claims of Pasteur and Koch should prove valid —no other contagious disease has been prevented by the elaboration of the brilliant idea of inoculation. Diseases were also checked by certain drugs which seemed to have the power of destroying their bacilli cause; but with these but two diseases had been checked in the history of medicine. Here was the field and it remained but for the student of to-day to work in it. There was everything to accomplish and the road was made clear before him. After formulating his suggestions in detail and giving illustrations of how genious had labored long to solve some of the simplest problems in life, the speaker said in conclusion:

The motto of the New York Academy of Medicine, coming down from mythological times, is this: "*Homines deos accedunt hominibus dando salutem.*" "Men most nearly resemble the gods when they afford health to their fellowmen." If, as seems now highly probable, Robert Koch should succeed in curing and preventing tubercular disease in its various manifestations, what greater reward could he possibly ask for than the pleasure which he must feel when the reflection to which this motto gives rise comes to his mind. The reward of the physician whose happy discovery stamps out a disease which before his day slew its thousands, comes from the hand of no Emperor, his glory from the appreciation of no applauding multitude, his renown from the pen of no fulsome historian. For him the victor's crown comes from the hand of the immortal God, his glory from the satisfaction of doing a great and glorious work, his renown from the gratitude of his fellowmen! The "great awakening light" which blessed the eyes of Abou Ben Adhem, not the imperial purple which decked the shoulders of the mighty Julius, constitutes his diadem and causes a halo to shine around his head! In the golden days of chivalry, when a young knight was to receive the accolade and become the defender of the weak and the redresser of wrong, he was required to spend an entire night in cathedral or other solemn place, reflecting upon the purity, the beneficence and the granduer of his new office, and in forming the noble resolve to make of it no trade, but to administer his duties with the of love man in his heart and the glory of God in his soul. Let this night and these exercises bear to you the relation of that vigil night of old! Medicine is the noblest of professions, the meanest of trades. Unless you can live lives of purity, of virtue, of honor and of honesty, seek a livelihood elsewhere and insult not the gods by striving through base methods and ignoble ambitions to resemble them. Will you not now fully agree with me when, in closing this address, I ask you whether the possibilities of medicine are not really greater than those of her sister sciences and arts? Will you not accord in my postulate that arms, arts, literature, science, all have their rewards, but that not one of them surpasses in the magnificence of its gifts those of which the god-like science, medicine, is capable? When, a quarter of a century hence, I meet with one of you, as we both wend our ways along the highway of life, my locks as white as the driven snow and yours as white as mine are now, come up to me, report yourself as a member of the graduating class of the Long Island College Hospital of 1891, and tell me which one of the beneficent discoveries which the next twenty-five years are sure to bring forth has been the means of causing you to resemble the gods and enrolled your name "among the few, the immortal names that were not born to die," and I, recalling at once to mind you and this pleasant evening which has made us acquanited, will bid you God-speed, even as I do to-night.

